# Analysis of Breast Cancer Information on Facebook Using Neural Network–Based Topic Modeling and Metadata Analysis of English and Spanish Content: Comparative Study

**DOI:** 10.2196/79161

**Published:** 2025-10-15

**Authors:** Rasika Muralidharan, Arthur D Soto-Vasquez, María Montenegro, Danny Valdez

**Affiliations:** 1 Luddy School of Informatics Indiana University Bloomington United States; 2 Hank Greenspun School of Journalism and Media Studies University of Nevada, Las Vegas Las Vegas United States; 3 Department of Spanish and Portuguese Rutgers University—New Brunswick New Brunswick, IN United States; 4 School of Public Health Indiana University Bloomington Bloomington United States

**Keywords:** breast cancer, cross-culture, digital health, Latinx, social media

## Abstract

**Background:**

Breast cancer is the most common cancer diagnosis among women, with approximately 2.3 million new cases annually. When faced with a cancer diagnosis, individuals often turn to the internet for information or reassurance, despite the risk of encountering low-quality or incorrect information. While this observation is well documented in English, limited work has been done to understand the quality of breast cancer information in Spanish, the second most commonly spoken language in the United States.

**Objective:**

This study uses natural language processing methods and quantitative modeling to analyze English and Spanish breast cancer posts from Facebook, a vital source of health-related information for 40% of English-speaking and 60% of Spanish-speaking adults in the United States.

**Methods:**

Using the CrowdTangle application programming interface, we collected and processed 243,029 English-language and 96,334 Spanish-language Facebook posts. We applied BERTopic with the all-MiniLM-L6 model and *k*-means clustering to infer thematic structures and used coherence scores to determine the optimal number of topics for each language. Descriptive statistics compared metadata differences across languages. We calculated descriptive statistics and ran inferential tests for likes, comments, and shares. Finally, we examined the top 1% (n=2430 English and n=963 Spanish) of the most engaged content to analyze differences in poster characteristics across languages.

**Results:**

Coherence scores indicated an optimal topic solution of *k*=40 (coherence=0.58) for English and *k*=30 (coherence=0.52) for Spanish. Thematically, we observed similar content in both languages, with topics spanning mammography, breast cancer events, pink ribbon month, and personal narratives. However, Spanish posts included local and municipal breast cancer events not present in English. Additionally, Spanish posts were more likely to mention at-home breast exams, which are no longer recommended in the United States. Engagement behavior showed statistically significant differences by language across likes, comments, and shares. English posts exhibited more consistent liking and sharing behavior, while Spanish posts showed more consistency in commenting. The top 1% (n=2430) of engaged content in English came from leading breast cancer nonprofits, whereas in Spanish (n=963, 1%), it originated from local governments or food and beverage companies.

**Conclusions:**

Facebook breast cancer content is generally consistent across languages. However, differences in engagement behavior suggest that English- and Spanish-speaking populations engage with content differently, highlighting cultural variability that warrants further exploration. Notably, leading cancer authorities may not have a strong presence in Spanish, indicating that the most accurate and up-to-date information may not be reaching a population particularly prone to worse breast cancer prognoses.

## Introduction

### Background

When faced with life-changing news of a breast cancer diagnosis—the most common cancer diagnosis among women globally—individuals often turn to the internet to search for information and reassurance [1]. In 2020, there were approximately 2.3 million new breast cancer cases, accounting for 11.7% of all cancer diagnoses worldwide in both men and women [2]. North America, which includes the United States, Canada, and Mexico, has some of the highest breast cancer prevalence rates, despite notably high 5-year survival rates [3]. However, in this region and especially in the United States, well-documented barriers to accessing medical care persist, which collectively impact breast cancer screening timing and breast cancer survivability. These barriers disproportionately impact vulnerable populations, including people with limited or no insurance, racial and ethnic minorities, immigrants, people who live in rural areas, and those with limited English proficiency [4].

Without regular access to or engagement with medical care, social media may become the primary, and in many cases exclusive, source of health-related information, including for information related to cancer diagnosis, treatment, and prognoses [1,5]. Yet, in an analysis of the 200 most popular social media posts related to breast, prostate, colorectal, and lung cancer, 32.5% were found to contain misinformation, and 30.5% were deemed harmful [6]. Posts classified as misinformation or harmful garnered significantly higher engagement than accurate content, highlighting the disproportionate reach of misleading cancer information on social media [7,8].

For Spanish speakers in the United States and abroad, Facebook has become a widely used platform for health-related information, with 80% of the Spanish-speaking population worldwide using it as a de facto internet and health-related search tool [9,10]. Though widely used, resounding evidence suggests that Spanish-language content on Facebook is often unreliable and more likely to include nonscientific remedies and outdated recommendations compared to English-language content. Exposure to social media content may have critical implications for health outcomes among Spanish speakers, as exposure to inaccurate information may influence health-related behaviors and decisions [11].

These concerns have gained attention in the public sphere [12]. A 2022 letter to Meta Platforms, Inc, from the US Senate highlighted significant gaps in their Spanish-language moderation, which is outsourced and has repeatedly failed to produce data and evidence on the effectiveness of these actions [13]. Furthermore, recent studies indicate that Facebook failed to flag 70% of incorrect Spanish-language health content, compared to only 29% in English [14]. Despite these findings, studies specifically analyzing the quality of Facebook health information are limited by small sample sizes and few have conducted comparative analyses by language.

To our knowledge, there are no large-scale analyses of breast cancer-related social media posts, nor are there comparative studies of breast cancer content in English and Spanish on social media platforms. This gap underscores a need to contextualize the role and impact of social media as a health information–seeking tool. This paper addresses this need by providing a comparative analysis of a large corpus, consisting of 347,085 Facebook posts related to breast cancer and breast cancer screenings, using natural language processing (NLP) and data mining methods. Our aim is to identify potential content differences across languages and to characterize the role of social media as a health information–seeking tool. Before we detail our study, an overview of breast cancer information–seeking behavior among English and Spanish speakers in both North and South America is warranted.

### Social Media as a Health Resource in the United States and Latin America

Recent studies suggest that 80% of US and 40% of Latin American adults regularly go on the internet to seek information about health care or medical issues [15]. In both regions, the internet and social media are widely regarded as strong community-building and social support tools to assist people who are facing difficult health challenges, including a cancer diagnosis [16]. However, beyond social support, evidence also routinely points to overreliance on the internet and social media to inform health beliefs about health issues, including cancer severity, cancer prognoses, and preventive cancer screening behaviors [17]. This overreliance is particularly evident among Spanish- and English-speaking Latina women in the United States, who are drawn to culturally resonant content on platforms such as Facebook. One recent study found that English- and Spanish-speaking Latino people in the United States gravitate to visual and food-related content on Facebook when searching for information about cancer, especially content promoting the curative properties of popular Latin American foods [15]. This calls for a much closer look.

In the context of breast cancer information available on Facebook, studies have noted some clear distinctions in content topics between languages. A study found that English-language breast cancer groups on Facebook tend to focus on awareness and fundraising more than social and emotional support [18]. While awareness groups have the most members, support groups have the most active contributors. In another case, a close qualitative study of an English-language breast cancer awareness Facebook page found users took conversations in their own direction, sometimes using comments to seek information from the wisdom of the crowd, which was not always accurate. The page did not moderate comments, posing some misinformation risk. The authors also observed that the posts were heavily gendered and focused on commodifying breast cancer. Indeed, a large scoping review of studies on national health organizations found their messages often focused on fundraising over actionable health information [19].

In contrast, for Spanish-language users, the availability of accurate health information remains a more significant challenge. For many Spanish-speaking users, social media often becomes the first and primary source of health information due to the lack of high-ranking, reputable health content in online search results (eg, Google) [20]. Thus, the first place they may find information about breast cancer is on social media rather than reputable websites. While previous studies have focused on English-language content [21], one recent analysis of social media analyses of vaccine information shows more misleading or incorrect health-related social media information in Spanish [22]. In sum, as Rivera and colleagues [23] argue, there is a “need for more robust models that conceptualize social media engagement, particularly as it relates to issues relevant to Latino/a communities,” especially given the moderation issues highlighted in the US letter to Meta.

This previous, primarily qualitative work has demonstrated that, across a variety of health-related conditions, Spanish-language content is sometimes outdated, not grounded in up-to-date science, or in some cases nonexistent [24]. These associations have not, however, been supported with larger sample sizes that greatly exceed qualitative norms. In a similar vein, computational studies of social media data—or studies leveraging data mining and algorithms to analyze large-scale data en masse—have similarly outlined clear limitations regarding the accuracy of social media content relative to scientifically supported advice for health or medical topics [25]. However, very few quantitative studies have been conducted in languages other than English, such as Spanish, despite evidence suggesting that information quality in other languages may vary more significantly due to less moderation than English-dominant feeds [26]. With research pointing to over 60% of Latino people trusting social media for health information across health topics [27], there is an urgency in identifying gaps in effective health communication on the internet.

NLP offers an opportunity to study large social media datasets. NLP is a scientific field and umbrella term of computer science and text-based data mining methodologies that use advanced mathematical computations to draw unique conclusions about structured and unstructured language data [28]. Historically, NLP has been applied in a variety of contexts, including to generate themes from a body of product reviews and social media posts [29], predict commonalities among documents in a corpus [30], characterize sentiment of social media posts [31], and, increasingly, to compare similar corpora stratified by a categorical variable, such as language [22].

As a methodology, NLP, and data mining more broadly, have made it possible to meaningfully analyze a large collection of social media posts simultaneously. Advances in computational modeling have further enabled the ability to process language data in languages other than English using a variety of open-access tools, including large language models and coding specific to the task of processing content in each language. However, the accuracy of these multilanguage tools varies due to design and implementation differences.

### Literature Gap and Study Rationale

Previous studies have captured the use of computational multilanguage comparative text analysis. Valdez et al [22] observed unique nuances by language in a collection of English and Spanish posts on X (X Corp), pertaining to vaccines. English content largely focuses on vaccine promotion, though pseudoscientific and politicized claims about vaccines and adverse health outcomes, including autism and heart attack, were observed. In Spanish, there was greater observed emphasis on vaccine distribution equity and mistrust of US- and European-based vaccines over China- and Russia-based vaccines, suggesting geographic and even geopolitical differences. While breast cancer and breast cancer screening content may be less prone to mistruths, pseudoscience, and misleading facts than the COVID-19 vaccines, given the politicization of them, we know people with questions about breast cancer may turn to the internet for such information. This warrants further insight into social media breast cancer content and disparities that may be attributed to language.

This study contextualizes the digital ecosystem on breast cancer and breast cancer screening content found on Facebook, a vital source of health-related information for almost 40% of English-speaking and 60% of Spanish-speaking adults residing in the United States [9]. Using NLP tools, we aim to capture the diversity of breast cancer–related dialogues on Facebook in English and Spanish (May 2023-2024). Our study is guided by the following research questions (RQs):

RQ1. What thematic differences emerge in a corpus of English- and Spanish-language Facebook posts pertaining to breast cancer?

RQ2. How do metadata metrics, including likes, comments, and shares, differ between English- and Spanish-language Facebook data about breast cancer and breast cancer screenings?

RQ3. What do the top pages receiving the most engagement through likes, comments, and shares imply about popular breast cancer Facebook content in English and Spanish?

Findings from this study will provide insight into the landscape of general breast cancer–related dialogues on Facebook, including themes and characteristics of highly engaged content. These insights will enhance our understanding of how Facebook, and social media more broadly, functions as a passive and intentional source of health information that is accessible to people regardless of potential language barriers. More importantly, these insights will potentially implicate differences in the quality of breast cancer and breast cancer screening information across languages.

## Methods

### Data Collection

Data for this comparative English- and Spanish-language study were collected between March 2023 and May 2024 using CrowdTangle, a third-party application programming interface with access to data from Instagram, Facebook, and other Meta properties. As one of the first studies to contextualize breast cancer and breast cancer screening content on Facebook in either English or Spanish, our aim was to capture a wide diversity of information that can be gleaned through simple searches on the platform. To identify our sample, we included a wide array of search queries comprising hashtags and other Boolean search strings that could be equivalently searched in English and their appropriate Spanish-language counterpart. In English, our queries included “breast cancer,” “Prevent breast cancer,” “mammography,” “mammogram,” and “Breast cancer screening.” We also used the accompanying hashtags “#breastcancer,” “#preventbreastcancer,” “#mammogram,” “#breastcancerscreening,” and “#breastcancerprevention.” Spanish-language queries were all equivalent translations of those used in English and included “cáncer de mama,” “prevenir el cáncer de mama,” “mamografía,” “mamograma,” “detección de cáncer de mama/seno,” and their accompanying hashtags “#cáncerdemama/seno,” “#prevenircáncerdemama/seno,” “#mamograma,” “#deteccióndecáncerdemama/seno,” “#prevencióndelcáncerdemama/seno.”

### Sample

After removing duplicates and posts that were observably not in English or Spanish, our sample size comprised 243,032 posts in English and 96,334 in Spanish, resulting in a composite sample size of 339,366 posts, collected between May 2023 and May 2024. Of note, as our study represented a holistic comparison by language, and language is often not specific to a certain region, we did not control for the nation of origin.

### Analyses

#### Exploratory Thematic Analysis With Word Clouds and Neural Network Topic Models

We used a language visualization tool based on term frequency—word clouds—to generate high-level insights into potential underlying themes in both English and Spanish texts. These visualizations served to cross-validate findings from neural network–based topic models. Topic models refer to an NLP task used to extract a fixed number of latent topics, or themes, from a large corpus of related texts [32]. For this analysis, we used BERTopic, a neural network–based topic modeling method that uses Bidirectional Encoder Representations from Transformers (BERT) to generate context-aware, semantically rich embeddings [33,34]. We selected BERTopic over more traditional models such as Latent Dirichlet Allocation (LDA), which relies on a probabilistic approach and lacks the ability to capture the contextual meaning of terms and their relationships. Additionally, previous research strongly suggests that BERTopic is well-suited for analyzing social media content and offers robust source-code documentation, enhancing reproducibility for future research even among those unfamiliar with NLP analyses [35,36].

#### Coherence Scores

To identify an optimal number of topics to extract from each dataset, we relied on coherence scores, which are a measure of topic quality [37]. We used a previously documented strategy, where we programmed the machine to iteratively run a topic model with an increasing number of topics (*k*=1-50) [37]. After each iteration, the computer also calculates a coherence score specific to that iteration. The iteration that returns the highest coherence score reflects an optimal number of topics, given the data. Although no clear cutoffs are salient in the literature, some research suggests that for noisy data, such as social media, coherence scores should range from 0.50 to 0.60, though coherence scores are known to vary significantly [38,39].

#### Analysis of Metadata and Engagement Data With Descriptive and Inferential Approaches

Metadata refers to information that is verifiable from a person’s profile, such as the post’s author, usernames, date and time of post, and other platform-specific characteristics. Metadata also includes engagement metrics per post, such as the number of likes, comments, and shares. When analyzed with descriptive and inferential approaches, metadata provides insights into the potential virality of the content and can specifically tell us what content resonates most with users, reflecting themes or content that appeal to people and groups of people.

### Procedure

**Figure 1:**
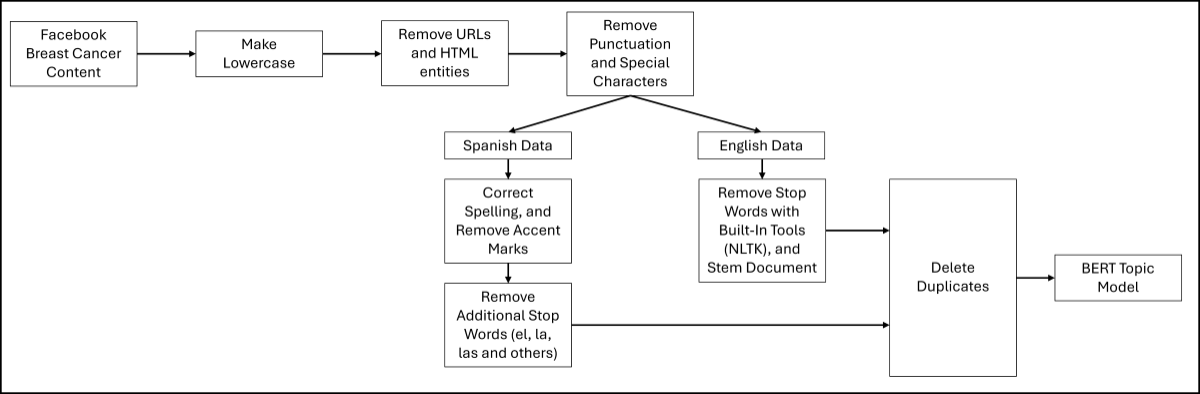
Flowchart offering a visualization of the data cleaning process for English- and Spanish-language text. Note that for English and Spanish, text cleaning processes were generally consistent. However, the Spanish text required additional preprocessing measures to eliminate additional stop words that are common in Spanish but not used in English (eg, de, la, es, and others). English language data was processed with standard packages including the Natural Language Toolkit (NLTK).

Our computational cleaning and analysis pipeline is depicted in Figure 1. We initiated our computational processes by preprocessing the text, which involved removing parts of speech that do not provide semantic meaning. As part of preprocessing, we removed URLs, HTML tags, account mentions, emojis, special characters, punctuation, and numbers. We also removed stop words in the English text, which include a fixed list of articles, prepositions, and commonly used words that generally do not provide context to the data (eg, it, the, and, of). Because our content is in both English and Spanish, it was essential to consider special steps when preprocessing in Spanish to account for the nuances of each language. We developed a second list of Spanish-language stop words (el, la, los, una, and others), which were removed from analysis. All accent marks were also replaced with standard English-language letters.

Once we preprocessed the data, we generated word clouds as an initial data exploration task. We then performed an iterative BERTopic model, using coherence scores as a model fit metric. As part of the pipeline, we applied *k*-means clustering to extract latent topics. The optimal number of clusters is determined by the highest coherence scores, which we calculated after every iteration, beginning with *k*=5 topics and *k*=50 topics in both English and Spanish. After running all iterations, the optimal number of topics—topic numbers with the highest coherence scores—for the English-language corpus was *k*=40 (coherence=0.58), while for the Spanish-language corpus, it was *k*=30 (coherence=0.52). All authors of the study contributed to an informal review of the topics to ascribe meaning to each topic and equally contributed to extracting highly representative posts per topic for further review.

Once we finalized our topic models in English and Spanish, we proceeded to analyze the metadata using descriptive and inferential tests. We used descriptive measures to capture patterns in overall engagement by calculating the means, medians, quartile ranges, standard deviations, and coefficient of variation (Cv) for likes, comments, and shares by language. We then tested for statistical differences in these engagement metrics using Mann-Whitney *U* tests, a nonparametric method appropriate for skewed data that violates assumptions of normality. To determine the appropriateness of the Mann-Whitney *U* test for our data, we visually assessed the distribution shapes of likes, shares, and comments by language using complementary cumulative distribution plots and compared distribution parameters using power-law fits. The distributions were deemed similar enough to justify Mann-Whitney *U* tests, which were then applied to likes, shares, and comments by language. Lastly, we extracted the most engaged content (n=2430 English and n=963 Spanish, top 1% of likes, comments, and shares) in both languages and compared them to test for differences in the frequency of certain page categories.

### Ethical Considerations

This study is a secondary analysis of deidentified, English and Spanish breast cancer content on Facebook. Given that no identifiable data were collected during the initial phases of the project, our study was deemed exempt by the Indiana University Institutional Review Board (#29108).

## Results

This computational study analyzed a collection of English- and Spanish-language breast cancer–related Facebook posts from March 2023 to May 2024. Using an NLP analysis pipeline, with a quantitative analysis of metadata, we effectively identified differences in English and Spanish content, which we highlight below without comment.

### RQ1. What Thematic Differences Emerge in a Corpus of English- and Spanish-Language Facebook Posts Pertaining to Breast Cancer?

We used word clouds to examine potential differences in language use by raw frequencies. Figure S1 in Multimedia Appendix 1 provides an overview of recurring unigrams (single word), bigrams (2-word clusters), and trigrams (3-word clusters). Larger words denote higher frequency in the data; smaller words denote lower frequency in the data. Recurring terms in either language include “breast cancer” (“cáncer de mama”), “early detection” (“detección temprana”), “women” (“mujeres”), and terms pertaining to “prevention” (“prevención”). While terms were similar in English and Spanish, we observed a key difference in the framing of cancer awareness month. In English, terms included (“awareness,” “month,” and “October”). In Spanish, terms were broader and more global (“día”/“day,” “mundial”/“global,” “internacional”/“international”).

**Figure 2:**
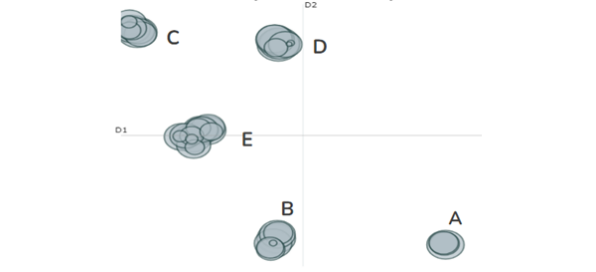
An intertopic distance map that visualizes the general similarity of topics extracted using BERTopic. This English-language intertopic distance map illustrates how topics extracted with BERTopic coalesced into 5 overarching clusters (cluster A: mammography; cluster B: breast cancer event and service promotion; cluster C: cancer support and outreach; cluster D: October and pink events; and cluster E: prevention and survivor support and awareness).

Figure 2 is a topic visualization of our English-language BERTopic model. This visualization highlights the total number of computer-identified topics (n=40) with circles ranging in size. In this figure, tightly grouped circles are considered hierarchical topics, meaning these topics are comprised of highly similar content in different contexts. For example, some circles in a grouped topic may detail how mammography works, while others that also discuss mammography may instead encourage women to receive breast cancer screenings. In contrast, circles that are further apart reflect content that is dissimilar from other topics. This figure depicts 5 clearly distinguishable clusters (grouped topics A, B, C, D, and E), each representing a general theme that is unique from the others.

Table 1 provides additional details about the hierarchical or grouped topics in English, including a cluster ID (derived from the analysis), grouped topic name, number of clusters per topic, number of posts sorted into a topic, and representative posts. From our grouped topics, topic E was the most prevalent (n=78,329, 32.2%). Upon reviewing several posts within this topic, we determined that this topic revolves around prevention, survivor outreach, and general breast cancer awareness. Topic A, which broadly addresses mammography, was the least representative in our data (n=20,268, 8%). Grouped topics B, C, and D appeared to refer to a variety of planned community events.

Regarding more nuanced thematic differences, we identified consistent and recurring patterns in the English data. In grouped topic A, for example, we identified a body of Facebook content focused on breast cancer screening promotion. As seen in quotations, “mammogram Xray images often find #breastcancer signs and symptoms early. Got questions? Read answers at #bcsm” and “early detection saves lives #grfresh #thinkfresh #choosefresh #breastcancer.” These messages largely encouraged mammography among Facebook users. Likewise, grouped topic B largely focused on breast cancer–related events, including fun runs, walks, and festivals. However, unlike grouped topic A, which seemed to largely focus on the United States, grouped topic B also included promotional content posted by clinics that appeared to originate from non-US countries, clinics and radiology laboratories located in other English-speaking countries, including the United Kingdom and many in Africa.

Grouped topic C represented content about events in support of breast cancer, such as school events, sporting events, and religious gatherings. Grouped topic D contained a more direct focus on early detection, mammograms, and support events for breast cancer awareness through social media events, giveaways, and apparel sales. The clusters under this topic also had several mentions of “pink,” “pink ribbon,” and other symbols denoting breast cancer awareness month: “October is breast cancer awareness month—purchase our hope bloom sunflower theme t-shirt today, use promo code at checkout #breastcancer #breastcancerawareness #workplacepro #hope #hopeforacure.”

**Figure 3:**
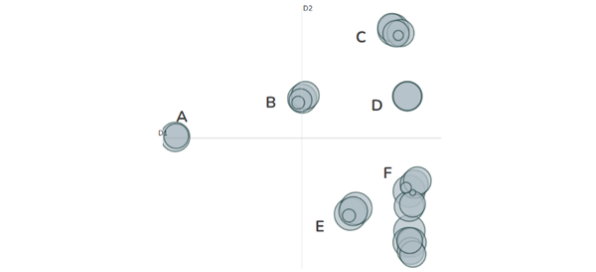
An intertopic distance map that visualizes the general similarity of topics extracted using BERTopic. This Spanish-language intertopic distance map illustrates how topics extracted with BERTopic coalesced into 6 overarching clusters (cluster A: mammography; cluster B: cancer types and potential treatments; cluster C: cancer support and outreach; cluster D: October and pink events; cluster E: prevention and survivor support and awareness; and cluster F: municipal, governmental, and other institutional support).

Figure 3 provides a visualization of our Spanish-language BERTopic model comprising 30 topics. As with the English-language findings, closely connected circles reflect similar content, while distal circles reflect different content. These topics were also grouped into overarching themes, or grouped topics, which comprised cluster IDs A, B, C, D, E, and one less distinguishable cluster ID F.

**Table 1:** Results from the BERTopic intertopic distance map for English, including a grouped topic column, clusters encompassing the grouped topic, the number and percentage of posts associated with each umbrella topic and cluster, and some representative posts from each umbrella topic. Cluster IDs refer to the exact topic number extracted from our BERTopic analysis and bear no effect on our results.

Topic ID	Umbrella topic	Cluster	Count (n=243,032), n (%)	Representative documents
A	Mammography	6, 23, 24	20,268 (8.34)	Regular mammograms are essential for detecting breast cancer. Experts recommend that women undergo mammograms every 1-2 years, and the benefits are substantial. Women who are at average risk for breast cancer should start mammogram screening at age 40 and get one every two years until age 74. That’s the latest updated recommendation from the U.S. Preventive Services Task Force (USPSTF), an independent, volunteer panel of national experts that makes recommendations focused on disease prevention Early detection is your greatest ally in identifying cancer at its earliest, most treatable stages. It can make all the difference in your breast health journey. By detecting potential issues early, you empower yourself with more treatment options and increase your chances of a successful outcome. Regular screenings, such as mammograms and clinical exams, are your proactive steps toward early identification.
B	Breast cancer event and service promotion	4, 5, 12, 25, 26, 27, 37	42,726 (17.58)	In support of Breast Cancer Awareness Month, Patel Hospital is offering 20% discount on Mammogram and Ultrasound Procedures throughout October 2023. We are delighted to have Dr Suresh, Medical Oncologist, Apollo Hospitals as a part of our “Thriving Beyond Diagnosis” session. Join us on 31st Oct, 8 PM, and gain the knowledge and support you need for a healthier, brighter future. Join us on Sunday, October 15th for the American Cancer Society: Making Strides Against Breast Cancer Walk!
C	Cancer support and outreach	2, 11, 17, 19, 20, 22, 32, 34	50,308 (20.7)	October is #BreastCancerAwareness Month–let’s come together to spread knowledge, support survivors, and work towards a world without #breastcancer. How are we helping support those with breast cancer? Well, we are doing what we do best, making candles! Through September and October we are selling our new ˜breast cancer candles and $1 from each will go to Ladies Fighting Breast Cancer, to help support survivors and those still on their breast cancer journey. Many thanks to Greece STORM Lacrosse for recently hosting their Stick it to Cancer fundraiser! Thanks to the players and their family and friends, the game raised $347 for the Coalition. We are inspired by their efforts to support survivors of breast and gynecologic cancers.
D	October and pink events	0, 1, 3, 10, 16, 30, 38, 39	51,401 (21.15)	Our 9th Annual Pink Ribbon Event is just around the corner! We can’t wait to see our guests all dressed up in PINK! A HUGE thank you to our donors and sponsors! This event SOLD OUT in record time! Beyond the pink ribbon: This March, join the conversation & help raise awareness for #BreastCancerRelatedLymphedema. Share resources, educate your network, & advocate for patient support. #BCRL #BreastCancer #Lymphedema #EmpoweringRecovery This month we are going PINK for Breast Cancer Awareness Month! Today, we delivered pink ribbon cupcakes to Staceys Bra & Lingerie. Stacey’s specializes in post mastectomy, compression and lumpectomy products as well as maternity and nursing bras. THANK YOU Stacey’s for not only supporting us, but our beautiful women throughout Iowa.
E	Prevention and survivor support and awareness	7, 8, 9, 13, 14, 15, 18, 21, 28, 29, 31, 33, 35, 36	78,329 (32.23)	Please allow us to introduce our 2023 speaker, Paige Lockton-Wilde. Mother, daughter, Olympic equestrian, her accomplishments are endless and breast cancer thriver belongs on the list. In The News: Patient Leticia Aguilar beat breast cancer for the second time and credits her recovery to the compassionate care received from our team. #CancerSurvivor #CancerWarrior CBS News’ Jennifer Bisram interviewed Ms. Aguilar who shared her story and encouraged all women diagnosed with #BreastCancer to, “Fight For Their Lives.” #CancerSurvivor Isabel shares her incredible story of resilience after a #BreastCancer diagnosis. Her experience highlights the importance of #StressManagement and emotional #support for #cancer patients from the moment of diagnosis and beyond. Learn about her story and how #MentalHealthMatters:

Table 2 illustrates that grouped topic F (municipal government) was the most prevalent grouped topic, comprising nearly 40% (n=41,786) of the entire Spanish-language sample. Upon reviewing several posts categorized in this group, we determined that the group largely focused on cancer outreach among local government entities. As with the English data, topic A in Spanish (mammography) was also the least prevalent grouped topic, comprising just 7% (n=6748) of the entire sample. Grouped topic B, positioned in the center of the intertopic distance map, refers to types of breast cancer and potential treatments by stage. Additionally, grouped topics C, D, E, and F (right-hand quadrants) collectively refer to myriad breast cancer events, including outreach, awareness, and support initiatives (Figure 3).

As observed in the English data, we documented that a significant portion of the discourse in this corpus addressed cancer prevention, treatment, and control in various contexts. Grouped topic 1 included posts originating from clinics and other medical entities promoting breast cancer screening services. As seen in the quotations, “La mamografía es un examen de prevención que todas las mujeres debemos conocer” (“Mammography is a preventive exam that all women should know about”) and “La #Mastografia o #Mamografia es un procedimiento que ayuda a toda mujer a encontrar anormalidades en las mamas que pueden desencadenar la presencia de #CancerdeMama” (“#Mammography or #Mastography is a procedure that helps all women detect abnormalities in the breasts that may lead to the presence of #BreastCancer”), these posts broadly refer to the importance of routine screenings.

Overlap in English and Spanish persisted in other topics. Grouped topic B, for example, comprised content broadly related to breast cancer, including outcomes, typologies, and prevention. Posts sorted into this topic recommended seeking medical attention after an observable symptom, while others provided guidance for individuals diagnosed with breast cancer to better understand tumor characteristics and treatment options. We also identified a wide body of posts pertaining to cancer awareness month (grouped topic C). Posts such as “¡Súmate al rosa! Apoyemos a todos los luchadores contra el cáncer de mama” (“Join the Pink movement! Let’s support all the fighters against breast cancer”) encouraged collective action and support for individuals currently fighting breast cancer. Grouped topics D and E focused on cancer prevention during Breast Cancer Awareness Month at the local level.

While we observed considerable overlap between the English- and Spanish-language content, differences also emerged. Compared with the English data, the Spanish-language posts placed greater emphasis on the importance of self–breast exams, which are no longer recommended in the United States but remain common and recommended practice in other Latin American countries (Banegas et al [40]). Emphasis in self-screenings is seen in quotations such as “¿CÓMO PREVENIR EL CÁNCER DE MAMA? Mantener un peso adecuado. Evitar el cigarro y el alcohol. Realizar una autoexploración mamaria mensual a partir de los 20 años, de preferencia al quinto día de la menstruación” (“HOW TO PREVENT BREAST CANCER? Maintain a healthy weight. Avoid smoking and alcohol. Perform a monthly breast self-examination starting at age 20, preferably on the fifth day of menstruation.”) Furthermore, and as mentioned, our Spanish-language data contained a greater presence of breast cancer–related promotions and events initiated by elected officials and political candidates. Yet despite these differences, the central focus in both the English- and Spanish-language data remained on breast cancer prevention and control.

**Table 2:** Results from the BERTopic intertopic distance map for Spanish-language, including a grouped topic column, clusters encompassing the grouped topic, the number and percentages of posts associated with each umbrella topic and cluster, and some representative posts from each umbrella topic. Cluster IDs refer to the exact topic number extracted from our BERTopic analysis and bear no effect on our results.

Topic ID	Umbrella topic	Clusters	Count (n=96,334), n (%)	Representative documents
A	Mammography	8, 23	6748 (6.99)	¡La mamografía salva vidas! (Mammograms save lives!) La #Mastografia o #Mamografia es un procedimiento que ayuda a toda mujer a encontrar anormalidades en las mamas que pueden desencadenar la presencia de #CancerdeMama. (#Mammography or #Mastography is a procedure that helps all women detect abnormalities in the breasts that may lead to the presence of #BreastCancer.)
B	Cancer types and potential treatments	9, 15, 24, 26	10,593 (10.97)	¡Los fibroadenomas son tumores benignos (no cancerosos) de las mamas, comunes y compuestos por tejido glandular y tejido estromal (conectivo)!→Son más frecuentes en mujeres de 20 a 39 años de edad, aunque pueden presentarse en mujeres de cualquier edad. (Fibroadenomas are common benign (non-cancerous) breast tumors composed of glandular and stromal (connective) tissue.→They are most frequent in women aged 20 to 39, although they can occur at any age.) SIGNOS Y SÍNTOMAS DEL CÁNCER DE MAMA Una parte importante de la salud de los senos consiste en saber cómo lucen y se sienten normalmente sus senos. El síntoma más común del cáncer de mama es una masa o bulto nuevo. Una masa no dolorosa, dura y con bordes irregulares tiene más probabilidades de ser cáncer, aunque los tumores cancerosos del seno también pueden ser sensibles al tacto, blandos y de forma redondeada. (SIGNS AND SYMPTOMS OF BREAST CANCER An important part of breast health is knowing how your breasts normally look and feel. The most common symptom of breast cancer is a new lump or mass. A mass that is painless, hard, and has irregular edges is more likely to be cancer, although breast cancer tumors can also be tender, soft, and rounded.)
C	Cancer support and outreach	3, 14, 17, 19, 28	15,310 (15.87)	La detección temprana es clave. Hagamos que la prevención sea un compromiso diario. Hoy, en el Día de la Concientización sobre el Cáncer de Mama, unámonos para apoyar a las valientes luchadoras. ¡Juntos hacemos la diferencia! (Early detection is key. Let’s make prevention a daily commitment. Today, on Breast Cancer Awareness Day, let’s unite to support the brave fighters. Together, we make a difference!) ¡Súmate al rosa! Apoyemos a todos los luchadores contra el cáncer de mama. #MuebleríaGuadalupe #Cuautitlán #OctubreRosa #CáncerDeMama (Join the pink! Let’s support all the fighters against breast cancer. #MuebleríaGuadalupe #Cuautitlán #PinkOctober #BreastCancer)
D	October and pink events	6, 12	7768 (8.06)	En el marco de las acciones del Mes Rosa para concientizar y sensibilizar sobre el #CáncerdeMama, este miércoles se dio a conocer el inicio de la campaña “Inspirando tu Salud”, a través de la cual se llevó a cabo la donación por parte de “Credinspira”. (As part of the Pink Month initiatives to raise awareness and promote understanding about #BreastCancer, this Wednesday marked the launch of the campaign “Inspiring Your Health”, through which a donation was made by “Credinspira”.) 19 de octubre: A nivel mundial, octubre es el Mes Rosa para crear conciencia sobre la detección oportuna. (October 19: Globally, October is Pink Month to raise awareness about early detection.)
E	Breast cancer prevention and awareness	0, 1, 11, 25	14,129 (14.65)	MADRES DE FAMILIA SE REALIZAN EXÁMENES PARA PREVENIR EL CÁNCER DE MAMA #Laredo | El día de hoy, 25 madres de familia Laredinas fueron beneficiadas con exámenes de mamografías para descartar cáncer de mama. (MOTHERS GET EXAMS TO PREVENT BREAST CANCER #Laredo | Today, 25 mothers from Laredo were provided with mammogram exams to rule out breast cancer.) ¿CÓMO PREVENIR EL CÁNCER DE MAMA? Mantener un peso adecuado. Evitar el cigarro y el alcohol. Realizar una autoexploración mamaria mensual a partir de los 20 años, de preferencia al quinto día de la menstruación. (HOW TO PREVENT BREAST CANCER? Maintain a healthy weight. Avoid smoking and alcohol. Perform a monthly breast self-examination starting at age 20, preferably on the fifth day of menstruation.)
F	Municipal, governmental, and other institution al support	2, 4, 5, 7, 10, 13, 16, 18, 20, 21, 22, 27, 29	41,786 (43.46)	Gobierno de Chilpancingo promueve acciones preventivas contra el cáncer de mama y cervicouterino ***Presidenta Norma Otilia Hernández Martínez pone en marcha la Clínica de Atención Integral para la Mujer Chilpancingo, Gro, a 25 de mayo de 2023.-“Queremos prevenir, queremos salvar vidas. Esta clínica de atención es para ustedes, para cuidar su salud”, dijo la presidenta. (Chilpancingo Government Promotes Preventive Actions Against Breast and Cervical Cancer ***President Norma Otilia Hernández Martínez Inaugurates the Comprehensive Care Clinic for Women Chilpancingo, Gro, May 25, 2023.-“We want to prevent, we want to save lives. This care clinic is for you, to take care of your health,” said the president.) Nombre de Dios.-La Presidenta del DIF municipal, Liliana Vázquez, junto a su equipo de trabajo, arrancó el día de hoy con el primer café rosa en la comunidad de Gabriel Hernández. Este evento es posible gracias a la Licenciada Marisol Rosso. (In the name of God-The President of the municipal DIF, Liliana Vázquez, along with her team, kicked off the first pink coffee event today in the community of Gabriel Hernández. This event is made possible thanks to Lic. Marisol Rosso.)

### RQ2. How Do Metadata Metrics, Including Likes, Comments, and Shares, Differ Between English- and Spanish-Language Facebook Data About Breast Cancer and Breast Cancer Screenings?

In addition to topic modeling analyses, we also examined differences in engagement metrics between our English- and Spanish-language datasets. Table 3 provides a summary of engagement metrics (ie, likes, comments, and shares) aggregated as means, standard deviations, and quartile ranges. Due to uneven sample sizes, we also calculated the Cv, a statistical measure calculated as the ratio of the standard deviation to the mean, for more effective cross-language comparisons.

**Table 3:** Comparison of engagement metrics (likes, comments, and shares) for English and Spanish Facebook data.

Measure		Likes	Comments	Shares
**English (n=243,032)**				
	n (%)	243,032 (100)	243,032 (100)	243,032 (100)
	Mean (SD)	43.20 (1004.09)	6.04 (73.40)	5.38 (100.99)
	Median (IQR)	4 (1-13)	0 (0-1)	1 (0-2)
	maximum	304,213	18,160	21,408
	Cv^a^ (%)	23.24	12.15	18.77
**Spanish (n=96,334)**				
	n (%)	96,334 (100)	96,334 (100)	96,334 (100)
	Mean (SD)	41.76 (631.73)	4.88 (89.02)	7.39 (80.94)
	Median (IQR)	5 (1-16)	0 (0-1)	1 (0-3)
	maximum	136,282	20,021	11,383
	Cv (%)	15.13	18.25	10.96

^a^Cv: coefficient of variance.

From Table 3, we observed that although the English dataset was significantly larger than the Spanish-language dataset, the aggregate means for likes, shares, and comments were generally similar. For English posts, the mean was 43.20 (SD 1004.09; Cv=23.24%; median 4, IQR 1-13) for likes, 6.04 (SD 73.40; Cv=12.15%; median 0, IQR 0-1) for comments, and 5.38 (SD 100.99; Cv=18.77%; median 1, IQR 0-2) for shares. For Spanish posts, the mean was 41.76 (SD 631.73; Cv=15.13%; median 5, IQR 1-16) for likes, 4.88 (SD 89.02; Cv=18.25%; median 0, IQR 0-1) for comments, and 7.39 (SD 80.94; Cv=10.96%; median 1, IQR 0-3) for shares. In both English and Spanish, the descriptive measures indicate a heavily right-skewed distribution for likes, comments, and shares, suggesting that only a small number of posts receive disproportionately high engagement across all 3 metrics.

Despite consistency in descriptive values, we observed differing variability in likes, shares, and comments by language. For likes, both datasets had similar observed means, but we observed that the Cv was higher in English (23.24% vs 15.13%). Higher Cv values indicate greater variability, suggesting a wider range of content that is highly engaged versus content that is not highly engaged. Similarly, while the average number of comments was higher in English, the Cv values indicated greater variability in the Spanish-language dataset (12.15% vs 18.25%), which suggests more consistent commenting behavior in English. Finally, in the Spanish-language dataset, we observed higher mean shares, with a lower Cv (18.77% vs 10.96%), which suggests Spanish posts were shared more consistently.

To assess whether descriptive differences were statistically significant, we performed a Mann-Whitney *U* test for each engagement metric by language as seen in Table 4. All comparisons were statistically significant, which confirms that engagement patterns, although descriptively similar, are apparently distinct across languages in our sample.

**Table 4:** Mann-Whitney U test results comparing engagement metrics like likes, shares, and comments by language. All comparisons were statistically significant at P<.05.

Engagement metric	Mann-Whitney *U* statistic	*P* value
Likes	11,497,783,887.50	<.001
Comments	12,535,136,172.00	<.001
Shares	11,263,541,515.00	<.001

### RQ3: What Do the Top Pages Receiving the Most Engagement Through Likes, Comments, and Shares Imply About Popular Breast Cancer Facebook Content in English and Spanish?

To better contextualize drivers of cross-language differences in engagement, we next examined the top 1% (n=2430 English and n=963 Spanish) of most engaged posts and their respective page categories. Page category, an end point in the CrowdTangle application programming interface, categorizes accounts based on their primary attribute, including whether the page identifies as a nonprofit organization, health clinic, doctor, or other. Table 5 provides the relative frequency of the most represented page categories in the English- and Spanish-language datasets. In English, the top 3 page categories included nonprofits, hospital pages, and for-profit charity organizations. In contrast, the top 3 page categories in Spanish were media news companies, news websites, or some type of governmental organization across a variety of levels. Especially unique in the Spanish-language data was the wider presence of politicians and content from local community pages (ie, community pages belonging to local communities, as opposed to English, where the pages were of communities that have a national presence), which was not observed in the English dataset.

After summarizing the frequency of various page categories, we aggregated the top 1% (n=2430 English and n=963 Spanish) engaged content based on relative likes, comments, and shares. We triangulated this information with respective page categories to further isolate who posted the most engaged content relative to page type, such as nonprofit organizations, for-profit charities, and other page categories.

**Table 5:** The most frequent page categories in English and Spanish on Facebook postings about breast cancer and breast cancer screenings.

Page category		Values, n (%)
**English**		
	Nonprofit	34,222 (14.67)
	Hospital	17,770 (7.62)
	Charity organization	11,798 (5.06)
	Media news company	7476 (3.2)
	Medical health	7343 (3.15)
	Government organization	5862 (2.51)
	News site	5686 (2.44)
	Community	4440 (1.9)
	Health site	4344 (1.86)
	Broadcasting	3585 (1.54)
**Spanish**		
	Media news company	20,051 (21.89)
	News site	8283 (8.99)
	Government organization	6120 (6.68)
	Hospital	3694 (4.03)
	Nonprofit	2908 (3.17)
	Topic newspaper	2598 (2.84)
	Politician	2292 (2.5)
	TV channel	2065 (2.25)
	Radio station	1863 (2.03)
	Local	1850 (2.02)

As seen in Table 6, in English, across likes, comments, and shares, the top 3 users with the most engaged content consistently included Breast Cancer NOW (@BreastCancerNow), the National Breast Cancer Foundation (@NationalBreastCancer), and Susan G Komen (@SusanGKomen). All 3 are registered on Facebook as nonprofit organizations focused on breast cancer research, outreach, and advocacy, though we recognize that the nonprofit status of non-US entities such as Breast Cancer NOW is somewhat unclear given differing requirements to be considered a nonprofit organization across countries.

In Spanish, the sources of the top 1% (n=963) of engaged content were more diverse and did not originate solely from nonprofit organizations. Instead, content came from health-related pages and websites (eg, @masalladlcancer, a Facebook page discussing products contaminated with ethylene oxide, a group 1 carcinogen), food and beverage companies (@AguasSanCarlosPeru), a physician (@LuizLizamaRodriguez, a cardiovascular doctor), and a politician (@MaraLezamaOfficial, the governor of the Mexican state of Quintana Roo). Only 2 breast cancer–specific organizations (BCSOs) were in the top 1% (n=963) for engagement in the comments category—@HistoriasDeSuperacion, an organization that encourages users to share their medical stories, and @OncoAyudaAC, which provides support to people diagnosed with cancer. Notably, neither of these 2 organizations appears to promote or distribute research related to breast cancer.

**Table 6:** Relative frequency of username and page category in the top 1% (n=2430 English and n=963 Spanish) of engaged content.

Engagement			Page category	Relative frequency, n (%)
**English**				
	**Likes**			
		@BreastCancerNOW	Nonprofit	155 (6.4)
		@NationalBreastCancer	Nonprofit	76 (3.2)
		@SusanGKomen	Nonprofit	38 (1.75)
	**Comments**			
		@BreastCancerNOW	Nonprofit	99 (4.2)
		@NationalBreastCancer	Nonprofit	47 (2)
		@SusanGKomen	Nonprofit	26 (1.15)
	**Shares**			
		@BreastCancerNOW	Nonprofit	55 (2.5)
		@NationalBreastCancer	Nonprofit	46 (2)
		@SusanGKomen	Nonprofit	36 (1.5)
**Spanish**				
	**Likes**			
		@masalladlcancer	Health site	14 (1.52)
		@AguaSanCarlosPeru	Food or beverage	8 (0.82)
		@MaraLezamaOfficial	Politician	7 (0.78)
	**Comments**			
		@HistoriasDeSuperacion	Nonprofit	23 (2.5)
		@OncoAyudaAC	Nonprofit	12 (1.32)
		@masalladlcancer	Health site	9 (1)
	**Shares**			
		@MaraLezamaOfficial	Politician	13 (1.75)
		@LuizLizamaRodriguez	Topic doctor	9 (1.42)
		@masalladlcancer	Health site	7 (1.4)

## Discussion

### Overview

Our findings support that Facebook, as one of many social media platforms that is highly accessed by the Latinx community, remains a key source of breast cancer–related knowledge in English and Spanish. However, observed differences in engagement across likes, comments, and shares, as well as differences in common page categories, warrant additional discussion.

### Thematic Similarities in English- and Spanish-Language Facebook Breast Cancer Data Suggest the Platform Is Broadly Used for Cancer-Related Support and Outreach

Our findings reflect that Facebook, as one of several leading social networking websites, contains diverse information pertaining to breast cancer and breast cancer screenings in English and Spanish. All components of our analysis, which included word clouds, topic models, and a metadata analysis, demonstrated a recurring pattern of cancer-related content that was categorized into breast cancer science, breast cancer advocacy, and breast cancer outreach. In English, much of this content was easily traceable to leading breast cancer authorities, such as Susan G Komen and the American Cancer Association. While content from those leading authorities was not as easily traceable in Spanish, we did note more observations pertaining to local and municipal breast cancer awareness promotion, which was not as apparent in our English-language findings. Importantly, in either language, we did not observe content in word clouds or topic models that appeared to contain blatantly incorrect or offensive content, though it is very likely that a more granular qualitative review might identify content not aligned with current breast cancer science.

These results in our English and Spanish analyses might imply, then, that for people with limited access to medical systems or questions about breast cancer, Facebook could serve several primary purposes. First, in English, exposure to Facebook content from Susan G Komen and other leading cancer authorities may encourage women to intentionally seek preventative screening measures, as has been documented previously with exposure to scientifically credible websites [41]. Likewise, the high presence of pink events, both in English and Spanish, can also facilitate social connection among women diagnosed or predisposed to breast cancer, breast cancer survivors, or caregivers of someone with breast cancer [42]. In Spanish, the higher levels of community outreach and breast cancer screening promotion may encourage women in a more convenient and comfortable setting, including through the promotion of local health fairs that offer low- or no-cost screenings [43]—strategies that have been previously leveraged in mobile or digital health interventions to promote screening behaviors in underserved populations [44]. We further acknowledge, however, that these findings are directly linked to Facebook and no other Meta Inc properties. We contend that it is likely content on other Meta platforms will differ substantially from Facebook due to unique target demographics.

### Highly Engaged in English- and Spanish-Language Content Reveals Disparities in Trusted Sources of Information

Both the English and Spanish data had heavily right-skewed distributions for likes, shares, and comments. This suggests that only a few posts garner significant attention in either language. However, upon extracting the top 1% (n=2430 English and n=963 Spanish) of content—that is, highly engaged in either language—further nuances emerged that warrant further context. In the English dataset, the top pages represented were nonprofits, charities, and hospitals devoted specifically to breast cancer care. Further, in the English corpus, the most frequent producers of content were the Susan G Komen Foundation, Breast Cancer Now, and National Breast Cancer—known breast cancer authorities and advocacy groups [45]. In contrast, most engaged content in Spanish came from non–breast cancer authorities, such as news outlets, entertainment, and political figures. The lack of dedicated organizations reflects the findings of Novillo-Ortiz and colleagues [20], which find a gap in reliable information on the internet in Spanish-speaking countries, particularly in Latin America. We see a similar effect here, a lack of state or nongovernment organization sources, and the gap filled by media companies and politicians.

However, previous studies have discussed how online, entertainment-focused media can have positive health benefits while conceding the potential for misinformation risk [46,47]. Yet, this is an underresearched area in non-English languages, and it is unclear how specific cultural constructs may amplify any exaggerations or mistruths. There is a need for deeper qualitative investigations, such as those called for by Hernandez and Martinez [48], into Spanish-language health communication aimed at Latino people or Latin American audiences. For example, we find that the most engaged Spanish-language content came from a range of sources that should provoke some caution. First, a health-adjacent page, @masalladlcancer, discussing products contaminated with ethylene oxide, a group 1 carcinogen. Second, a food and beverage company, @AguasSanCarlosPeru. Lastly, 2 individuals with tangential expertise on breast cancer, a cardiovascular doctor, @LuizLizamaRodriguez, and a politician, @MaraLezamaOfficial, the governor of the Mexican state of Quintana Roo. This discrepancy raises important concerns for public health because nonspecialist sources may not always provide scientifically accurate or up-to-date information about breast cancer prevention, screening, and treatment. While such platforms might still play a role in raising awareness, the lack of expertise and medical grounding could lead to misinformation or inadequate health guidance for the audience. It is beyond the scope of this exploratory paper to examine why the content they create about breast cancer is engaging to Spanish-speaking audiences, but it is worth a closer look in future work, including specific insights into breast cancer screening recommendations and the extent of alignment with current recommendations across North and South America.

### Differences in Engagement Between English- and Spanish-Language Facebook Breast Cancer Data Highlight Nuanced Social Media Use Styles Among English- and Spanish-Speaking Audiences

In addition to these previous observations, differences in engagement behavior—measured by Cv and substantiated with inferential tests—manifested in sharing, commenting, and liking behavior differences in either language. In English, the Cv was higher for likes and shares; however, in Spanish, we observed a higher Cv for comments. This suggests more variation in liking and sharing behavior in English and more variation in commenting behavior in Spanish. In terms of engagement implications, metrics such as likes and shares could be viewed as more superficial compared to comments, which may reflect deeper cognitive engagement with content [49]. Cv for English likes and shares may suggest that English-speaking audiences engage less deeply, favoring content driven by virality and visibility rather than liking or sharing all content equally. In Spanish, liking and sharing were consistent; however, we observed greater variability in commenting, which might suggest that the type of content may predict commenting behavior more effectively in Spanish. Given that the most engaged content in English originated from leading nonprofits, we can reasonably conclude that content containing higher levels of likes and shares (and possibly comments) in English originated from these major nonprofits. These results suggest that content that invites interaction and discussion may drive engagement for Spanish-speaking users, while virality and the identity of the poster may be more important for English-speaking audiences.

### Limitations

This study has limitations that we hope to address in future work. First, due to the limitations of CrowdTangle’s scraping capabilities, our dataset represents only a small portion of the total volume of breast cancer–related content on Facebook, whether in English or Spanish. While we believe our work is representative of the broader Facebook breast cancer ecosystem, our findings may not be fully generalizable to all breast cancer content on Facebook or on other social media platforms. Second, our metadata analysis focused exclusively on “likes,” despite Facebook offering several other engagement options, such as “sad,” “angry,” “funny,” and “caring.” We emphasized “likes” because it is the closest analog to engagement metrics used on other social media platforms, such as Meta’s Instagram, which only offers a “like” option. Third, our thematic analysis was entirely unsupervised, meaning no training data were used to generate the findings, aside from the mini large language model accompanying the BERTopic library. While additional training data would likely yield more refined topics, we believe the exploratory nature of our RQs warranted the use of equally exploratory tools for data mining. Future research should consider replicating our study with more refined models, potentially using our data to help build such infrastructure.

### Conclusions

This study used NLP techniques to analyze English- and Spanish-language Facebook posts related to breast cancer. Our principal findings reflect that the platform serves as an important source of information in both languages. Topics in either language encompassed breast cancer care, screening advocacy, and cancer-related outreach. However, the origin of the content differed extensively. In English, the most engaged content linked to authoritative cancer organizations like Susan G Komen. In Spanish, the most engaged content showed more entertainment- and politically oriented pages. These findings highlight Facebook’s role as a valuable information hub and also underscore the need for enhanced scrutiny of trusted sources in non-English health communication on the internet. As such, the clearest recommendation of our study is that BCSOs, such as the Susan G Komen Foundation, need a much stronger presence on social media in Spanish.

Our findings also suggest that a tailored approach to digital interventions is strongly warranted. In English, it may be sufficient to ensure that leading authorities in breast cancer maintain a social media presence. However, for Spanish-language social media, a more nuanced approach that considers cultural differences may be needed. Indeed, storytelling and culturally resonant intervention methods are more effective in promoting health behaviors among marginalized populations [50]. The cultural value of familismo, or duty to family, may also be an effective digital communication tactic [22]. As seen in our data, commenting was more varied in Spanish; thus, the need to identify content that drives helpful commenting behavior, such as sharing narrative accounts of breast cancer journeys, may potentially be more impactful than messaging from leading cancer organizations. We strongly recommend additional qualitative work that examines social media use in the context of familismo—specifically, in a new media and breast cancer context—to understand how differing members of the family use social media for health purposes.

Future research should also consider detailed qualitative research with these data to gain deeper insights, especially into popular pages in Spanish-speaking communities. Such studies could potentially investigate why existing Spanish-language content from official organizations, such as Susan G Komen, is either nonexistent or is failing to gain currency with target audiences. Previous work offers valuable insights into cancer-related content and could serve as a model for qualitative English and Spanish breast cancer social media research [17,23]. Second, we contend that expanding our data collection to include other more popular platforms, such as Instagram and TikTok, will provide a more comprehensive understanding of the disparity in English and Spanish breast cancer content and can capture nuance that may be attributed to differing target demographics. Finally, using methods that assess the impact of social media on communities, such as interviews, surveys, and other community-oriented methods, is equally important and necessary to understand the impact of social media on screening intentions among Spanish-speaking women.

## Appendix

Multimedia Appendix 1 Word clouds derived from our English and Spanish breast cancer Facebook data accounting for unigrams, bigrams, and trigrams.

## Abbreviations

BCSO: breast cancer–specific organization
BERT: Bidirectional Encoder Representations from Transformers
Cv: coefficient of variation
LDA: Latent Dirichlet Allocation
NLP: natural language processing
RQ: research question

## Data Availability

Data and source code used in the study are available in a secure GitHub repository upon reasonable request.
