# Supplementary material for: Analysis of Breast Cancer Information on Facebook Using Neural Network–Based Topic Modeling and Metadata Analysis of English and Spanish Content: Comparative Study
Source: J Med Internet Res. 2025 Oct 15;27:e79161. doi: 10.2196/79161 (PMC12572747; doi:10.2196/79161)

**Figure S1.** This visualization showcases word clouds derived from our English- and Spanish-language breast cancer Facebook data, accounting for unigrams, bigrams, and trigrams. These terms reflect their frequency, where larger words denote more observations and smaller words denote fewer observations. From the word clouds, we observed high overlap between languages.


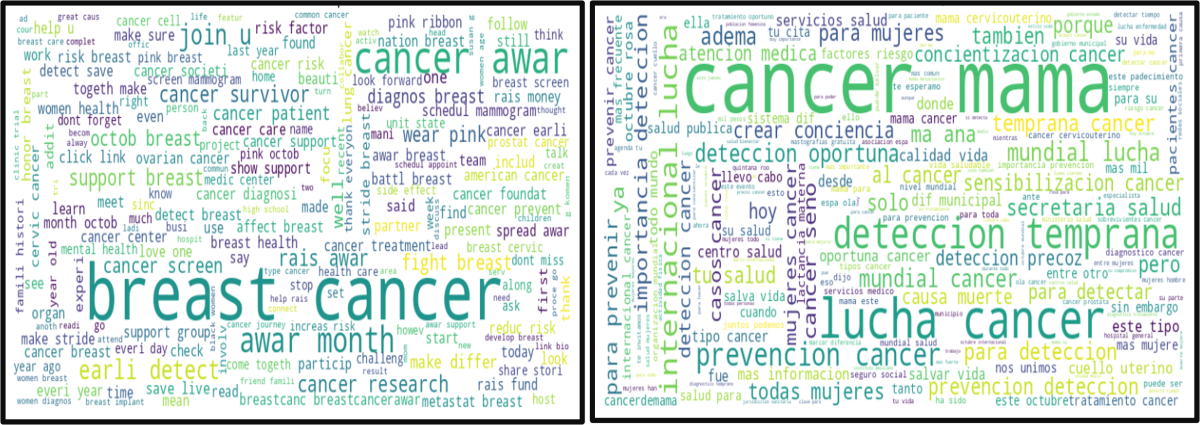

Supplement: Multimedia Appendix 1 [file jmir_v27i1e79161_app1.docx]
